# “AND” or “OR” logic operations of DNA probes: Fluorescent detection and discrimination of ovarian cancer cells via dual-microRNA in complex environments

**DOI:** 10.1016/j.mtbio.2025.102454

**Published:** 2025-10-23

**Authors:** Guanghui Wang, Yuting Li, Shuangjie Liu, Jing Li, Meizhen Yao, Jiaxiang Cheng, Ting Chen, Jia Zhang, Fenglei Gao, Wensheng Du, Lei Hua

**Affiliations:** a Department of Obstetrics and Gynecology, Affiliated Hospital of Xuzhou Medical University, Xuzhou, Jiangsu, 221000, China; b Department of Obstetrics and Gynecology, Suining People's Hospital, Xuzhou, Jiangsu, 221200, China; c Department of Neurosurgery, The Affiliated Hospital of Xuzhou Medical University, Xuzhou, 221002, China; d Jiangsu Key Laboratory of New Drug Research and Clinical Pharmacy, Xuzhou Medical University, Xuzhou, Jiangsu, 221004, China

**Keywords:** DNA logic gate, miRNA detection, Identification of ovarian cancer cells, Fluorescence detection

## Abstract

The development of DNA logic circuits has enabled novel signal carriers for the identification of cancer cells. Addressing the limitations of single-target detection in ovarian cancer, including insufficient specificity and signal delay, this study innovatively constructs a dual-miRNA detection system based on “AND” or “OR” logic operations. In the “AND” logic pathway, both miR-221 (LOD is 0.1792 nM) and miR-96 (LOD is 0.1990 nM) are required to cooperatively trigger strand displacement amplification (SDA) for fluorescence signal activation. In contrast, the “OR” logic pathway enables signal output through either miR-221 or miR-96 (LODs are 0.1020 nM and 0.056 nM, respectively), significantly enhancing sensitivity for low-abundance samples. The core innovation lies in the hairpin structure-mediated miRNA recycling mechanism, where competitive binding allows target molecules to repeatedly participate in reactions, thereby amplifying signal efficiency. This strategy demonstrates high specificity in SKOV3 cells, and its modular design enables rapid adaptation to other miRNA combinations (e.g., miR-21/miR-155) through DNA sequence reprogramming, offering a versatile platform for multi-tumor subtyping. The approach provides new perspectives for intelligent, portable biosensing and bioanalytical applications.

## Graphical abstract

A dual miRNA detection strategy was proposed based on "AND" or "OR" logic operations for ovarian cancer cell identification.

**Figure:**
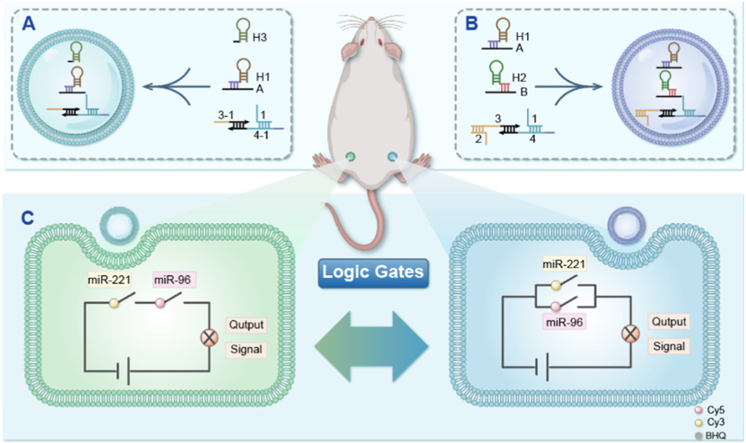

## 1 Introduction

Ovarian cancer is one of the most commonly diagnosed malignancies worldwide, posing a significant threat to women's health. Due to its complex pathogenesis, unclear early symptoms, and relatively poor prognosis, early diagnosis of ovarian cancer remains a significant challenge. Therefore, early and rapid detection of specific biomarkers is crucial for improving diagnostic accuracy. Among these biomarkers, microRNAs (miRNAs) are endogenous, non-coding small RNA molecules that play key roles in regulating gene expression, cell differentiation, and apoptosis [[1], [2], [3]]. The abnormal expression and dysregulation of certain miRNAs can modulate signaling pathways related to other target genes, thereby influencing disease progression. For example, miR-221 and miR-222 induce TRAIL resistance by targeting the tumor suppressors PTEN and TIMP3, and enhance cell migration by activating the AKT pathway and metallopeptidase [4]. MiRNA-96 is overexpressed in ovarian cancer cell lines, while its downregulation reduces the proliferation and migration capabilities of these cells by targeting FOXO3a [5]. Similarly, miR-221 is highly expressed in ovarian cancer cell lines and has been shown to target the oncogene KIT and the tumor suppressor p27kip1 [6,37]. Based on these findings, numerous research groups have developed biosensors for miRNA in situ fluorescence imaging. For instance, Yang et al. [[7], [8], [9]] employed DNA as a vector to deliver siRNA and various compounds into cells for tumor therapy and imaging; Li et al. [10,11] developed a range of DNA nanodevices for tumor detection; and Wang group [12] designed a multifunctional, stimulus-responsive, self-catalytic hybridization component circuit to achieve effective miRNA imaging in live cells and mice. However, these technologies primarily focus on detecting a single miRNA in individual ovarian cancer cells. Due to limited sensitivity and specificity, relying on a single biomarker for ovarian cancer detection is suboptimal, especially in complex biological environments where single miRNA detection can lead to limited practical applications and an increased risk of false positives. Therefore, the simultaneous detection of multiple miRNAs is expected to enhance clinical applicability.

Logic gate circuits process and relay digital signals, and their sophisticated design plays a crucial role in detecting and analyzing multiple biomarkers within complex biological environments [13]. Researchers have constructed a variety of bio-logic circuits for a wide range of applications in various fields such as biosensing [14,15], drug delivery [16,17], and cell behavior control [[18], [19], [20]]. DNA, as a common biomolecule in cells, has excellent biocompatibility, specificity, and programmability, so it can be designed into various forms of signal carriers and skillfully applied in the field of biosensing [[21], [22], [23]]. However, the traditional methods, such as Northern blot and qRT-PCR are susceptible to the interference of homologous miRNA family cross-reaction. The application of catalytic hairpin self-assembly [24,25], hybridization chain reaction [26,27], entropy-driven strand displacement amplification [28,29] and rolled-circle amplification [30,31] is also gradually developing and maturing. Traditional DNA logic circuits use specific markers such as metal ions [32], enzymes [33], and nucleic acids [34] as input signals, which are converted into signals as outputs through a series of reaction processes. A DNA logic module was integrated, providing digital YES and NO outputs to reflect the multiple input signals from various biomarkers. This design meets the essential criteria for a “quick screening” diagnostic tool and benefits from the programmability and simplicity of the logic module, making it adaptable to a wide range of applications.

**Scheme 1:**
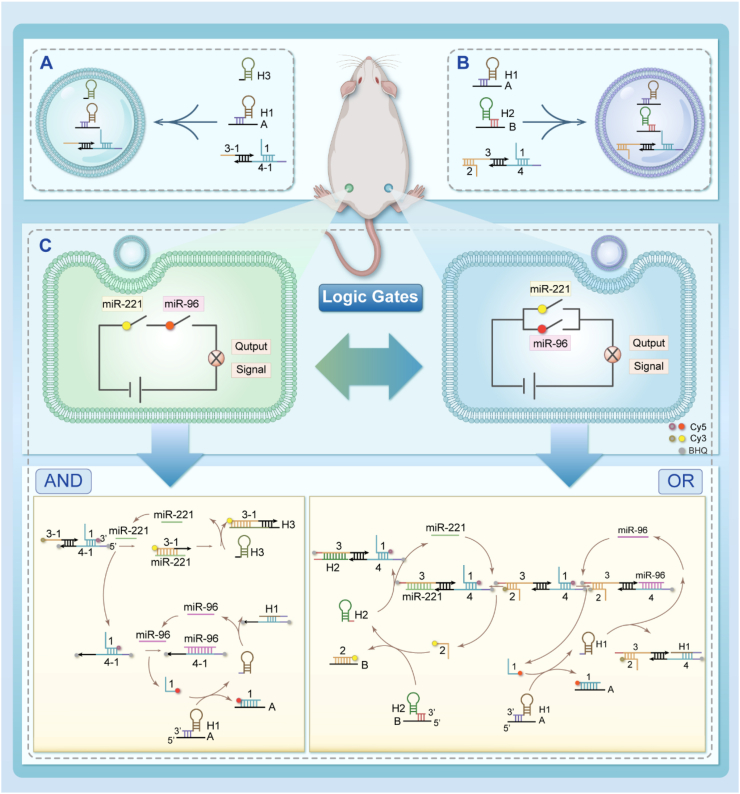
(A) Assembly diagram of AND logic circuit; (B) Assembly diagram of OR logic circuit. (C) The operation process of AND and OR logic circuits.

Although DNA logic gate technology is relatively well-developed, its application in multi-target detection for ovarian cancer has not yet been fully integrated. Therefore, there is an urgent need to develop a dual-target microRNA detection platform based on DNA logic operations that can accurately identify ovarian cancer cells and facilitate potential clinical applications. In this study (Scheme 1), we designed a DNA logic circuit to detect two miRNAs, miR-221 and miR-96, in SKOV3 cells. The circuit includes an AND gate that can simultaneously detect two miRNAs and an OR gate that can independently detect both miRNAs. The AND logic circuit comprises a pre-assembled detection probe (DP) and two hairpin structures (AH1, H3). In the AND logic circuit, the DP probe binds to miR-221 and miR-96, which are highly expressed in ovarian cancer cells, triggering a strand displacement reaction. Subsequently, these two hairpin structures competitively bind to the miRNAs, allowing the miRNAs to re-enter the cycle. If fluorescence is generated during detection, it is encoded as “1”. If no fluorescence is detected, it is encoded as “0”. The OR logic circuit also comprises a pre-assembled detection probe (DP2) and two hairpin structures (AH1, BH2); however, the detection of miR-221 and miR-96 does not interfere with one another, producing distinct output signals. Utilizing two distinct logic circuits effectively addresses the issues of poor stability, limited sensitivity, and low specificity in single-target detection, enabling accurate differentiation between ovarian cancer cells and normal cells. This work integrates DNA logic gate technology with highly expressed miRNAs in ovarian cancer, offering novel insights for clinical diagnosis, therapeutic research, and prognostic detection.

## 2 Experimental section

### 2.1 Assembly of DNA logic circuits

Pre-customized DNA single strands were solubilized with nuclease-free sterile water at an initial concentration of 100 μΜ. All DNA single strands were heated in a thermostatic metal bath for 5 min (at 95 °C) and then slowly cooled to room temperature. The required single chain was fully mixed in the ratio of 1:1, so that the final concentration was 1 μM, and incubated at 37 °C for 3 h.

### 2.2 Confocal laser scanning microscopy imaging analysis

SKOV3 cells, A549 cells, and HeLa cells were grown using DMEM medium containing 10 % fetal bovine serum at 37 °C, 5 % CO_2_, and 95 % relative humidity. The transfection experiment was carried out according to the reagent instructions. The cells were seeded into 6-well plates and grown in the incubator. The experiment was carried out when the cell density reached 70–80 %. After the waste liquid was poured out, 2 mL of fresh complete medium (including 10 % FBS) was added for standby. Mix 20 μL of DNA (1 μM) with 2 μL of Lipo 8000™ transfection reagent in 125 μL of DNEM medium (without serum or antibiotics), mix thoroughly, and incubate at room temperature for 6 h to stabilize. Finally, 125 μL of mixed solution was added to each well of cells and continued to culture for 24 h. Discard the waste liquid. It can be detected by washing three times with PBS solution.

### 2.3 In vivo imaging

All animal experiments involved were approved by the Experimental Animal Center of Xuzhou Medical University and were performed according to the guidelines for use and care. SKOV3 cells were injected subcutaneously into the right hind limb site of Female BALB/c nude mice to obtain the corresponding tumor model. The DNA transfection complex was prepared by combining 20 μL of DNA (1 μM) with 2 μL of Lipo8000™ transfection reagent in 125 μL PBS, followed by thorough vortexing and incubation for 6 h at room temperature. Subsequently, 30 μL of the mixture was injected into tumor-bearing mice. The mice were anesthetized with isoflurane at different time periods, and then were imaged in vivo. First, adjust the concentration of isoflurane to 2–4 %, fill the induction box with gas, and then put the mice into the induction anesthesia. After 2–3 min, the mice were removed and switched to the mask to maintain anesthesia, and the concentration was adjusted to 1–2 %. After complete anesthesia, it was placed on the imaging platform for in vivo imaging analysis.

## 3 Results and discussion

### 3.1 Design of and logic circuits and or logic circuits

In the AND logic circuit (Scheme 1C left), DP is pre-assembled from the 1-strand, 3-1-strand, and 4-1-strand and delivered transiently into the cell along with AH1 and H3. Upon entry of the DNA probe into the cytoplasm, miR-221 binds first to the 3-1 strand, while the 3-1 strand disassociates from the 4-1 strand, releasing Cy3 fluorescence and exposing the miR-96 response site. Then miR-96 replaces the 1-strand and binds to the 4-1-strand to show Cy5 fluorescence. Due to the low intracellular miRNA abundance, we additionally designed two hairpin structures (H1 and H3) for releasing miRNAs back into circulation for intracellular fluorescence imaging. DP2 in the OR logic circuit (Scheme 1C right) is composed of 1 chain, 2 chain, 3 chain, and 4 chain, both sides are symmetrical structures. Therefore, the detection of miR-221 and miR-96 do not interfere with each other and produce one-to-one output signals. MiR-96 (miR-221) reacts with the 4 (3) chain in a chain displacement reaction, releasing the 1 (2) chain to produce the corresponding fluorescent signal. 1 (2) chain binds with the A (B) chain to release H1 (H2) and the 4 (3) chain binds, releasing the miR-96 (miR-221) to re-enter the detection circuit. Before performing the experiments, we used NUPACK software (which can be used for the analysis of nucleic acid structures) to analyze the predesigned DNA strands in a predictive manner. Fig. S1 shows the combination of DP, where the three strands are combined in an orderly manner according to the pre-designed structure. Fig. S2 shows the structure of DP2, with symmetrical structures on both sides, while the binding site for the strand displacement reaction is reserved, which is consistent with the prediction. Fig. S3 shows the structure of AH1 and BH2.

### 3.2 Feasibility analysis of DNA logic circuits

**Fig. 1:**
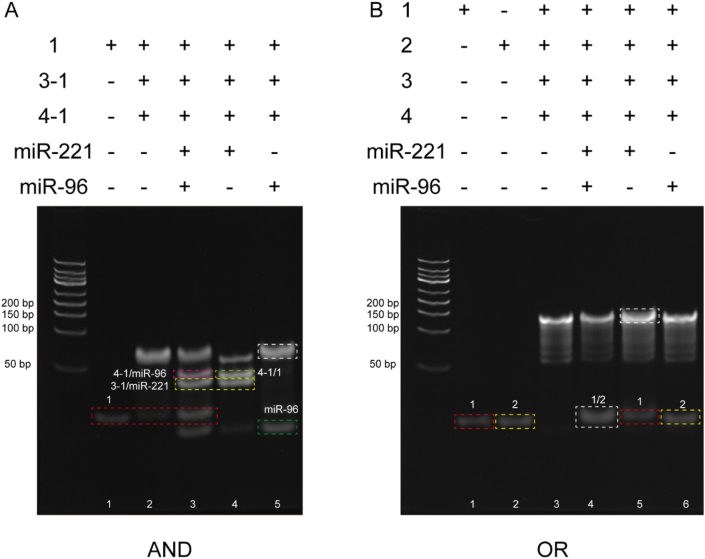
Polyacrylamide gel electrophoresis of targets mixed with different DNA logic circuits. The slowest migration strip is in the white box.

After annealing the DNA single strands at 95 °C for 5 min, the DNA probes were assembled by mixing the different DNA strands according to the designed Scheme 1. Fig. S4 shows the polyacrylamide gel electrophoresis of the single strands in the logic circuits, and the sizes of the DNA single strands were all below 50 bp. In Fig. 1A, channel 2 is the assembled DP reactant and is significantly larger than 50 bp, no excess bands were generated. This indicates that the three DNA strands were assembled successfully. In channel 3, we added miR-221 and miR-96, and the appearance of 1 strand can be observed. Two new bands appeared at 50 bp, which we could identify as 3-1/miR-221 and 4-1/miR-96. In channels 4 and 5, we added miR-221 and miR-96, respectively. From the results, there was no 1-strand band in channel 4, but two bands appeared at 50 bp. Only the band to miR-96 appeared in channel 5. From this, we can speculate that the addition of only one target in the AND logic circuit does not complete the whole pathway. Polyacrylamide gel electrophoresis of the OR logic circuit is shown in Fig. 1B. The assembled DP2 reactant is shown in channel 3 and can be seen to be larger than 100 bp. MiR-221 and miR-96 were additionally added in channel 4, while the 1-strand and the 2-strand appeared at the corresponding positions. The addition of miR-221 and miR-96 to channels 5 and 6, respectively, also resulted in the appearance of substituted 1- and 2-strands in the corresponding positions. The abundance of intracellular miRNAs is very low, usually at pM level. In order to solve this problem, we set up a cycle part in the logic gate, so that miRNAs can be reused, thereby increasing the fluorescence intensity. As shown in Fig. S5, H1 (H2) is replaced by 1 chain (2 chain) through chain displacement reaction. Subsequently, H1 replaced miR-96 to re-enter the cycle. Similarly, the H3 chain replaced miR-221 to re-enter the cycle.

**Fig. 2:**
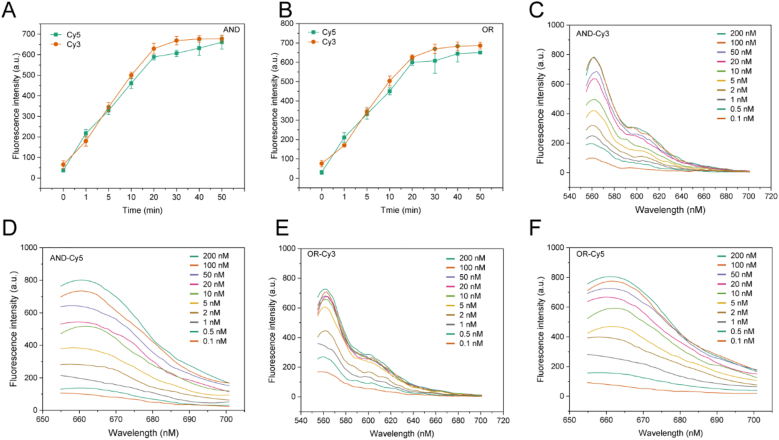
(A) Fluorescence intensity of a DNA strand after different times of reaction with the target strand in an AND logic circuit. (B) Fluorescence intensity of DNA strands after reacting with the target strand at different times in the OR logic circuit. (C) Fluorescence spectra of different concentrations of miR-221 after reaction with the AND logic circuit. (D) Fluorescence spectra of different concentrations of miR-96 after reaction with the AND logic circuit. (E) Fluorescence spectra of different concentrations of miR-221 after reaction with the OR logic circuit. (F) Fluorescence spectra of different concentrations of miR-96 reacted with the OR logic circuit.

**Fig. 3:**
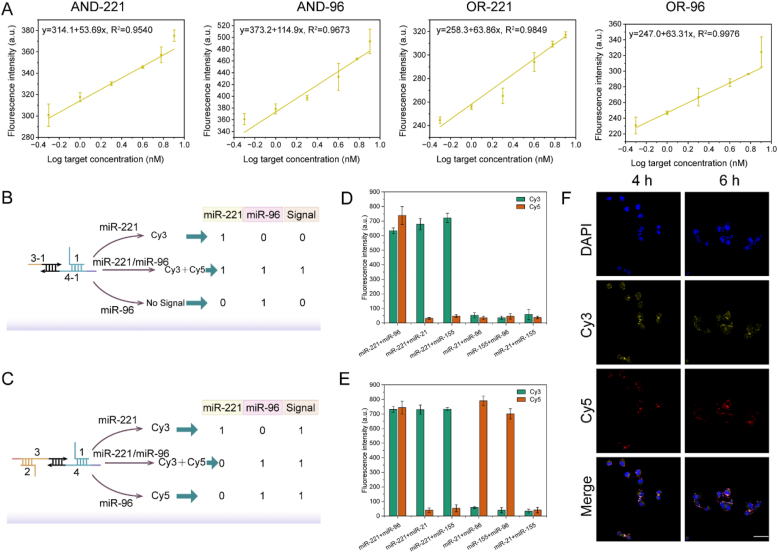
(A) The relationship between fluorescence signal (y) and target concentration (x). Error bars represent the standard deviations of three replicate measurements (0.5 nM, 1 nM, 2 nM, 4 nM, 6 nM, 8 nM). (B) Scheme diagram of “AND” logic circuit detection. (C) Schematic of OR logic circuit detection. (D) Diagram of in vitro specificity analysis of “AND” logic circuit. (E) Diagram of in vitro specificity analysis of “OR” logic circuits. (F) Confocal fluorescence imaging graph of SKOV3 cells after co-incubation with “OR” logic circuits for different times (Scale bar: 60 μm).

As shown in Fig. 2A and B, the fluorescence signals of both AND and OR logic circuits reached the maximum after 20 min of reaction, and thereafter there was no significant enhancement. On this basis, the feasibility of the DNA logic circuits was further evaluated using fluorescence spectra. Different concentrations of miRNAs were added to the reaction system, and the reaction was carried out for 20 min after sufficient mixing, and the fluorescence signals of both Cy3 and Cy5 were detected by fluorescence spectrophotometer, and the results are shown in Fig. 2C–F. The results showed that the fluorescence signals of both logic circuits increased with the increase in miRNA concentration. This indicates that after the addition of miR-221 and miR-96, the two logic circuits successfully performed logical operations and generated corresponding output signals. Then, in the AND system, 100 nM miR-96 was added first, and the corresponding miR-221 (0.5 nM, 1 nM, 2 nM, 4 nM, 6 nM, 8 nM) was added to the tube according to the pre-designed concentration gradient. The fluorescence spectra of the AND system for the target miR-221 in the low concentration range are shown in Fig. 3A, with a good correlation between fluorescence signals and logarithms of target concentrations (Log) showing a good linear relationship in the range of 0.5 nM–8 nM. In the above manner, we sequentially detected miR-221 and miR-96 in the AND system and OR system (Fig. 3A). According to the 3σ (3 times the standard deviation of the blank fluorescence signal) principle, the detection limits of miR-221 and miR-96 in the AND system were 0.1792 nM and 0.1990 nM, respectively, and those of miR-221 and miR-96 in the OR system were 0.1020 nM and 0.056 nM, respectively, demonstrating that the system has a certain detection potential in the assay.

### 3.3 Biosafety analysis and qRT-PCR

The biosafety of the DNA probe was analyzed before delivering the DNA probe into the cells. SKOV3 cells were incubated for a total of 12 h with the DNA probe after apposition and then stained by adding Annexin V-FITC/PI staining reagent. As shown in Fig. S6, the red fluorescence (dead cells) in the PI channel was negligible and the number of live cells reached more than 90 %. The incubated cells were digested down for flow cytometry results evaluation. The results are shown in Fig. S7, and the number of viable cells in the Q3-3 quadrant was all above 90 % as well. This proves that the DNA probe is well-biocompatible. Before using DNA probes for detection, we first used the traditional method (one-step RT-qPCR SYBR Green Kit) to verify the basic expression level of miR-221/miR-96 in different cells. As shown in Fig. S8, the relative expression levels of miR-221 and miR-96 in SKOV3 cells were much higher than those in hose-b cells, about 5 and 13 times higher than those in hose-b cells. The relative expression levels of miR-221 and miR-96 in OVCAR-3 cells were about 4 and 7 times higher than those in HOSE-B cells. However, the traditional qRT-PCR needs to design primers independently, and it is vulnerable to the interference of homologous miRNA family cross-reaction. Before doing PCR experiments, the cells must be lysed, and the spatio-temporal information cannot be retained. The DNA detection probe can monitor the dynamic changes of miRNA in real time without cell lysis.

### 3.4 Selectivity of dual-input logic circuits

As shown in Fig. 3B, in the AND logic circuit, if only miR-221 or miR-96 exists in the reaction system, the final output (Cy5) is “0”; only when miR-221 and miR-96 coexist, the output is “1”. In the OR logic circuit (Fig. 3C), the DP2 reactant is a symmetric structure, and the output is “1” when either or both miR-221 and miR-96 are present. The intracellular biological environment is complex, and miRNAs are not only in low abundance but also have very high similarity. Therefore, we additionally prepared other miRNAs to verify the selectivity of the logic circuit for other miRNAs. MiR-21 and miR-155 are two common miRNAs in the cell, so they are very suitable as interference terms. The results are shown in Fig. 3D and E and the output signals are consistent with the results of the truth table (Fig. 3B and C), regardless of the “AND” logic circuit or “OR” logic circuit. In the cycle part of the logic circuit, the hybrid region of A/H1 and B/H2 should not be too stable; otherwise, it is not conducive to the replacement reaction of 1 or 2 chains (Fig. S9). SKOV3 cells were transferred to confocal dishes and placed in a cell culture incubator for growth. 24 h later the probe was delivered into the cytoplasm using liposome transfection. Confocal fluorescence images showed that the fluorescence signals of Cy3 and Cy5 were maximal after 4 h of reaction, and did not enhance significantly after 6 h (Fig. 3F).

**Fig. 4:**
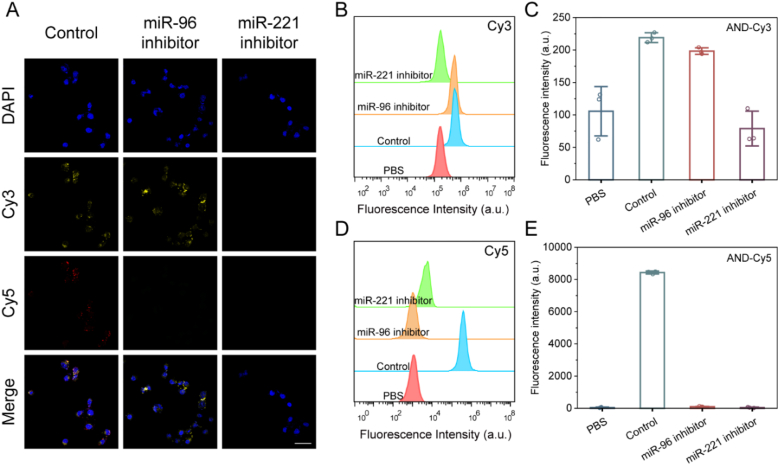
(A) Confocal fluorescence imaging image of AND logic circuit after co-incubation with SKOV3 cells. (B) Flow diagram of AND logic circuits after co-incubation with SKOV3 cells (Cy3). (C) Average fluorescence intensity values for each group in Figure (B). (D) Flow graph of AND logic circuits after co-incubation with SKOV3 cells (Cy5). (E) Average fluorescence intensity values for each group in Figure (D). (Scale bar: 60 μm).

**Fig. 5:**
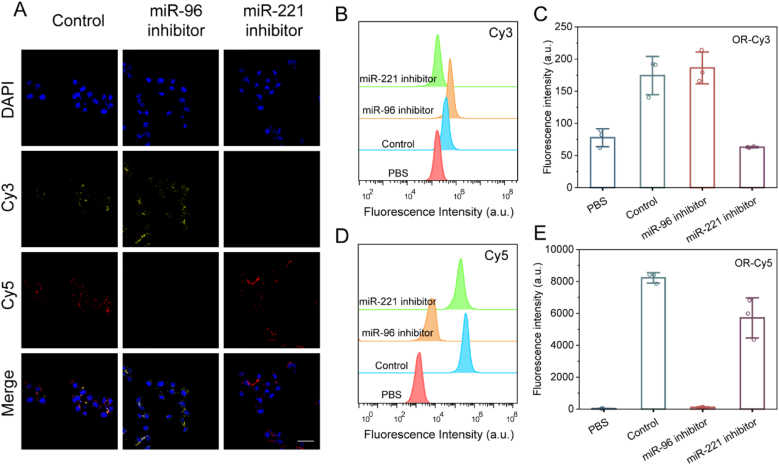
(A) Confocal fluorescence imaging image of OR logic circuit after co-incubation with SKOV3 cells. (B) Flow diagram of OR logic circuits after co-incubation with SKOV3 cells (Cy3). (C) Average fluorescence intensity values for each group in Figure (B). (D) Flow graph of OR logic circuits after co-incubation with SKOV3 cells (Cy5). (E) Average fluorescence intensity values for each group in Figure (D) (Scale bar: 60 μm).

To verify the arithmetic process of the DNA logic circuit, we co-incubated SKOV3 cells with miR-221 inhibitor and miR-96 inhibitor to simulate both (1, 0) and (0, 1) inputs, respectively. Fig. 4A shows the confocal fluorescence imaging of AND logic circuits. The control group was incubated with untreated SKOV3 cells with AND logic circuits, and two types of fluorescence, Cy3, and Cy5, could be observed after the reaction. MiR-96 inhibitor group was in (1, 0) input mode, and only Cy3 fluorescence, but not Cy5 fluorescence appeared. The miR-221 inhibitor group, on the other hand, was in the (0, 1) input mode, which did not expose the miR-96 linkage site in the absence of miR-221 presence and therefore did not show any fluorescence. Fig. 4B–E shows the flow plots and the corresponding average fluorescence intensity values, which are consistent with the confocal results. The OR detection system was grouped in the same way as the AND detection system. As shown in Fig. 5, the corresponding output signals appeared in both the miR-96 and the miR-221 inhibitor groups, which indicated that the OR logic operation results were executed, consistent with the expected results. However, no significant fluorescent signal was observed in L929 cells and HOSE-B cells (Fig. S10 and Fig. S11). The above results indicate that the probe has good target selectivity.

**Fig. 6:**
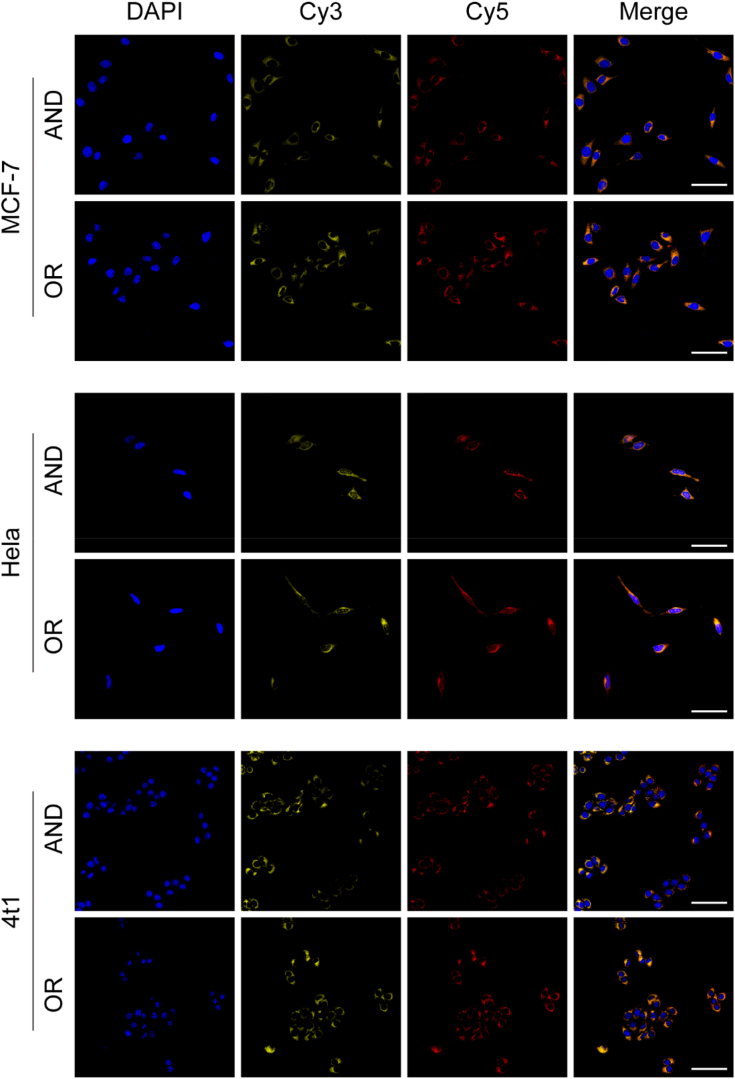
Confocal fluorescence imaging of DNA logic circuits after co-incubation with MCF-7 cells, HeLa cells, and 4T1 cells (Scale bar: 60 μm).

The DNA sequences are programmable, which facilitates our application of the DNA probes to the detection of miRNAs in other cells. Therefore, we can apply the DNA probes to other cells by simply replacing the complementary sequences of miR-221 and miR-96 in the DNA probes with the complementary sequences of the target detectors. We selected two cells, MCF-7 cells (miRNA-21, miRNA-155), 4T1 cells (miRNA-21, miRNA-155) and Hela cells (miRNA-21, miRNA-155) [35,36], for validation. As shown in Fig. 6, both fluorescent signals were evident from the confocal results. This indicates that the detection system was successfully executed according to the logical algorithm after being delivered into the cells.

### 3.5 Identifying specific tumor cells in vivo using DNA logic circuits

**Fig. 7:**
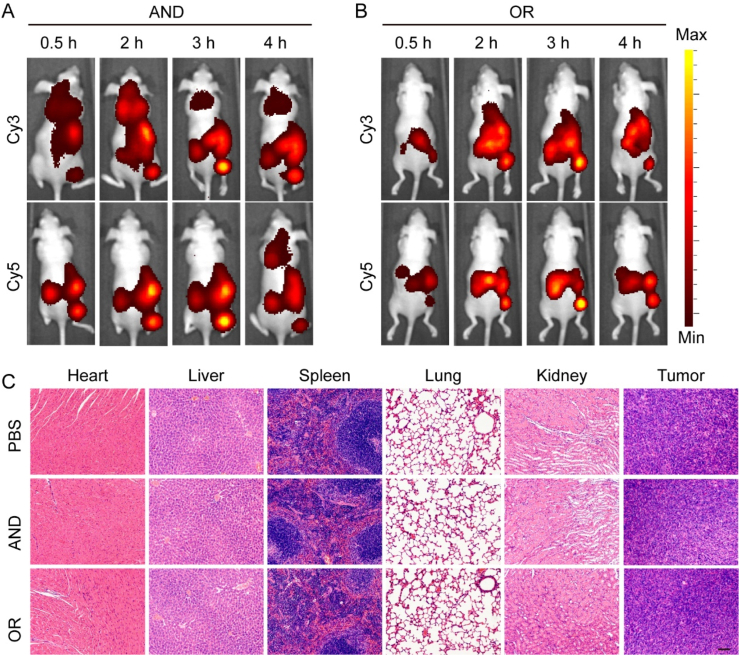
SKOV3 tumor-bearing nude mice. (A) *In vivo* fluorescence imaging images at different times after injecting “AND” logic circuit into a mouse. (B) *In vivo* fluorescence imaging maps at different times after injecting “OR” logic circuits into mice. (C) He matoxylin and eosin staining of major organs and tumors (heart, liver, spleen, lung, kidney, and tumor) in mice. (Scale bar: 200 μm).

The above in vitro experiments and cell experiments have effectively demonstrated the feasibility and reliability of two detection systems. Under these conditions, we further explored the in vivo application of the detection systems in mice. The detection systems were delivered within the tumor by liposome transfection. After the “AND” and “OR” detection systems were injected into SKOV3 hormonal nude mice, respectively, whole-body fluorescence images of mice were obtained at different time points. As shown in Fig. 7A, the fluorescence intensity of the tumor site in mice treated with the AND detection system gradually increased and reached the maximum at 3 h. The fluorescence intensity of the tumor site in mice treated with the AND detection system increased gradually and reached a maximum at 3 h. Within 4 h after injection, the fluorescence intensity of the tumor site was significantly greater than that of other normal tissues, confirming that the AND detection system has a detection function in mice. Fig. 7B shows the whole-body fluorescence images of mice processed by the OR detection system. The fluorescence intensity of the tumor site in mice increased.

with time and was significantly greater than that of other normal tissues. OVCAR-3 is an ovarian cancer cell line, and miR-221 and miR-96 are also up-regulated [5,37]. Like SKOV3 tumor-bearing nude mice, “AND” and “OR” detection systems were injected into OVCAR-3 tumor-bearing nude mice. As shown in fig. S13 and fig. S14, compared with other tissues, the fluorescence intensity at the tumor site was significantly the highest at 3h after injection, and gradually metabolized at 4H. It has been proven that the probe can effectively detect the up-regulated target miRNAs in mice. To assess the fluorescence response of normal organs and tumors, mice were euthanized 4 h after injection, and vital organs and tumors were collected for ex vivo imaging and H&E staining. As shown in Fig. S12 and Fig. 7C, no obvious lesions were found in the hearts, livers, spleens, lungs, kidneys, and tumor tissues of the mice, further demonstrating the biosafety of the DNA probe. Meanwhile, the tumor tissues contained residual fluorescent signals, some of which were metabolized out of the body through the liver and kidney. The above results indicate that the “AND” and “OR” detection system was successfully applied to the imaging analysis of tumor cells in vivo in mice. The probe was safe and did not damage the important organs of the mice.

## 4 Conclusions

In this study, we successfully integrated DNA logic gate technology with highly expressed miRNAs in ovarian cancer, constructing two detection modes, “AND” and “OR”, which accurately identified specific miRNAs in ovarian cancer cells. The introduction of a hairpin structure significantly enhanced the output signal, which is critical for the practical application of intracellular miRNA detection. This DNA logic detection system addresses the challenge of miRNA similarity by selectively detecting miRNA specificity. Furthermore, owing to the programmability and ease of operation of the logic modules, the “AND” and “OR” detection modes and the design principles of DNA strand displacement reactions can be flexibly applied. This adaptability allows for the extension of the detection system to other cellular and clinical applications. This system can simultaneously detect two types of miRNAs in ovarian cancer cells, amplify the detection signals. Therefore, we believe that this strategy holds significant potential for cancer cell type recognition and future clinical applications.

## CRediT authorship contribution statement

**Guanghui Wang:** Formal analysis, Data curation, Conceptualization. **Yuting Li:** Funding acquisition, Formal analysis, Data curation. **Shuangjie Liu:** Investigation, Data curation, Conceptualization. **Jing Li:** Formal analysis, Data curation. **Meizhen Yao:** Formal analysis, Data curation. **Jiaxiang Cheng:** Formal analysis, Data curation, Conceptualization. **Ting Chen:** Formal analysis, Data curation. **Jia Zhang:** Formal analysis, Data curation. **Fenglei Gao:** Visualization, Validation, Supervision. **Wensheng Du:** Validation, Supervision, Software. **Lei Hua:** Visualization, Supervision.

## Declaration of competing interest

The authors declare that they have no known competing financial interests or personal relationships that could have appeared to influence the work reported in this paper.

## Data Availability

Data will be made available on request.
