# Supplementary material for: “AND” or “OR” logic operations of DNA probes: Fluorescent detection and discrimination of ovarian cancer cells via dual-microRNA in complex environments
Source: Mater Today Bio. 2025 Oct 23;35:102454. doi: 10.1016/j.mtbio.2025.102454 (PMC12630042; doi:10.1016/j.mtbio.2025.102454)
Supplement: Multimedia component 1 [file mmc1.docx]

**Supporting Information**

**“AND” or “OR” Logic Operations of DNA Probes: Fluorescent Detection and Discrimination of Ovarian Cancer Cells via Dual-microRNA in Complex Environments**

Guanghui Wang^1,2,3,⊥^, Yuting Li^1,⊥^, Shuangjie Liu^1,⊥^, Jing Li^1^, Meizhen Yao^1^, Jiaxiang Cheng^1^, Ting Chen^2^, Jia Zhang^2^, Fenglei Gao^4,^*, Wensheng Du^1,^* and Lei Hua^1,3,^*

1. Department of Obstetrics and Gynecology, Affiliated Hospital of Xuzhou Medical University, Xuzhou, Jiangsu 221000, China.
2. Department of Obstetrics and Gynecology, Suining People’s Hospital, Xuzhou, Jiangsu 221200, China．
3. Department of Neurosurgery, the Affiliated Hospital of Xuzhou Medical University, Xuzhou 221002, China.
4. Jiangsu Key Laboratory of New Drug Research and Clinical Pharmacy, Xuzhou Medical University, Xuzhou, Jiangsu 221004, China.

^⊥^G. Wang, Y. Li and S. Liu contributed equally to this work.

*Corresponding Author. Email: hualei1012@aliyun.com (L. Hua), [dws11@126.com](mailto:dws11@126.com) (W. Du), jsxzgfl@sina.com (F. Gao).

**Contents**

[**Experimental Sections** S3](#_Toc170052932)

**The sequence of DNA (**[**Table S1)** S5](#_Toc170052933)

**The sequence of the MiRNA primer (**[**Table S2)** S6](#_Toc170052933)

**Structure of DP reactants (**[**Figure S1)** S7](#_Toc170052934)

**Structure of DP2 reactants (**[**Figure S2)** S8](#_Toc170052935)

**Structure of AH1 and BH2 (**[**Figure S3)** S9](#_Toc170052936)

**Gel electrophoresis of DNA single strand (**[**Figure S4)** S10](#_Toc170052937)

**Gel electrophoresis of cyclic chains (**[**Figure S5)** S11](#_Toc170052938)

**Cell death (**[**Figure S6)** S12](#_Toc170052940)

**Apoptosis (**[**Figure S7)** S13](#_Toc170052940)

**qRT-PCR (**[**Figure S8)** S14](#_Toc170052942)

**In vitro fluorogram (**[**Figure S9)** S15](#_Toc170052939)

**L929 cell fluorescence imaging image (**[**Figure S10)** S16](#_Toc170052941)

**HOSE-B cell fluorescence imaging image (**[**Figure S11)** S17](#_Toc170052941)

**Organ fluorescence images of SKOV3 tumor-bearing mice (**[**Figure S12)** S18](#_Toc170052942)

**In vivo fluorescence image of OVCAR-3 tumor-bearing mice (**[**Figure S13)** S19](#_Toc170052942)

**Organ fluorescence images of OVCAR-3 tumor-bearing mice (**[**Figure S12)** S20](#_Toc170052942)

**Experimental Sections**

**Materials and reagents.** Nanopore water (18.2 MΩ·cm) was used in all experiments. Dulbecco’s modified Eagle’s medium (DMEM), trypsin-EDTA digestion solution, One Step RT-qPCR SYBR Green Kit, and 4, 6-Diamidino-2-phenylindole (DAPI) were purchased from KeyGEN BioTECH. All fluorescence measurements were performed on a Hitachi FL4600 fluorescence spectrophotometer (Kyoto, Japan). Fluorescence microscopy images were recorded using an *FV10i (Olympus, Japan). Flow cytometric analysis was performed on a FACSCalibur flow cytometer (Becton Dickinson, USA). Gel electrophoresis was performed on a PowerPac HC electrophoresis analyzer (Bio-Rad, USA) and imaged on a Tanon-1600R gel imaging system (Shanghai, China) under UV irradiation. In vivo, fluorescence imaging was recorded on LB983 NightOWL II* Imaging System. (Berthold, Germany).

All oligonucleotides were synthesized and purified by Sangon Biotechnology Co. Ltd. (Shanghai, China), their sequences are shown in Table S1.

**8% nondenaturing polyacrylamide gel electrophoresis:** Wash and dry all slides beforehand. Place the two slides tightly together in the gluing trough, taking care to align the lower ends. Place the glue-making tank on the table, fill the space between the slides with deionized water, and let it stand for more than 20 min. If the water level does not drop significantly, the seal is good. The gel solution was configured during the resting period. Add 10.54 mL of deionized water, 4 mL of 5 × TBE buffer, 5.4 mL of 30% acrylamide, 100 mL of 10% AP (ammonium persulfate), and 4 μL of TEMED (tetramethylethylenediamine) to a 50 mL centrifuge tube and gently mix well. Pour out the deionized water in the middle of the slide and absorb the excess water with filter paper. Fill the slits in the center of the slide with the gel solution and insert the sampling comb vertically without creating air bubbles. The mixture is allowed to solidify for 40 min. Remove the gel from the table, place it in the electrophoresis tank, and fill it with 1 × TBE buffer starting from the center. Finally, 10 μL of 1 μM DNA strand solution was added and electrophoresed at 100 V for 45 min.

**Annexin V-FITC/PI double staining for apoptosis detection:** SKOV3 was inoculated into a six-well plate and grown until confluency. Add 125 μL of DNA probe solution and incubate with the cells for 12 h. Remove the waste solution, wash it three times with saline add the prepared working solution, put it into the incubator, and incubate for 30 min away from light. Discard the waste solution and add the appropriate amount of saline. Finally, cell death was observed using an inverted fluorescence microscope.

**Apoptosis experiments:** After co-incubating the cells with the probe, the cells were digested using EDTA-free trypsin. The collected supernatant was mixed with the digested cells and centrifuged, the cells were collected and washed twice with PBS. The dye was added and incubated at room temperature for 5-15 min and then the samples were uploaded for detection. All animal experiments were approved by the Animal Protection and Ethics Committee of Xuzhou Medical University. The ethical code of animal study is 202306T020.

**Inhibitors of transfection:** Transfection experiments were performed using Lipo8000™ Transfection Reagent according to standard procedures. Subsequently, the waste solution was removed and added to the appropriate detection system for autofluorescence imaging.

**Table S1.** The oligonucleotide sequences used in this work.

| **Oligonucleotides** | **Sequences (5’-3’)** |
| --- | --- |
| **miR-221** | **AGC UAC AUU GUC UGC UGG GUU UC** |
| **miR-96** | **AAU CAU GUG CAG UGC CAA UAU G** |
| **miR-21** | **UAG CUU AUC AGA CUG AUG UUG A** |
| **miR-155** | **UUA AUG CUA AUC GUG AUA GGG GUU** |
| **1** | **CCC AGG TTC TCT TTG CCA ATA TGC CC** |
| **2** | **GGG AGG TTC TCT TTG CTG GGT TTC** |
| **3** | **AAC CTC CCG AAA CCC AGC AGA CAA TGT ACG TGC TTTTTTTTTTTTTTT** |
| **4** | **AAC CTG GGC ATA TTG GCA CTG CAC ATG ATT CCC CC AAAAAAAAAAAAAAA** |
| **3-1** | **GAA ACC CAG CAG ACA ATG TAC GTC GCA TTC CCC TGC AG** |
| **4-1** | **AAC CTG GGC ATA TTG GCA CTG CAC ATG ATT CCC ACG TAC ATT GTC** |
| **H1** | **TGG CAC AAA ACT ATG TGC AGT GCC AAT ATG CCC AGG TT** |
| **H2** | **CCA GCA TTA CGA CAT TGT CTG CTG GGT TTC GGG AGG TT** |
| **H3** | **TGC GAC GTA CAT TGT CTG CTG GGT TTC GAC AAT GT** |
| **A** | **TTTTT CAT ATT GGC AAA GAG AAC CTG GGA TAT A** |
| **B** | **ATA TAG AAA CCC AGC AAA GAG AAC CTC CCA TAT A** |

**Table S2.** MiRNA primer sequence used in this paper.

| **miRNA** | **Sequences (5’-3’)** |
| --- | --- |
| **miR-221-F** | **GTT GGT GGG AGC TAC ATT GTC TGC** |
| **miR-221-R** | **GTG TCG TGG ACT CGG CAA TTC** |
| **miR-96-F** | **TTA GCT CAG GAT CAT CAT CAT TTA CAT AGA TAG GG** |
| **miR-96-R** | **AAC ACT CGA GTG AGA GAA GAG AGT GCC TAG A** |
| **U6-F** | **CTC GCT TCG GCA GCA CA** |
| **U6-R** | **AAC GCT TCA CGA ATT TGC GT** |


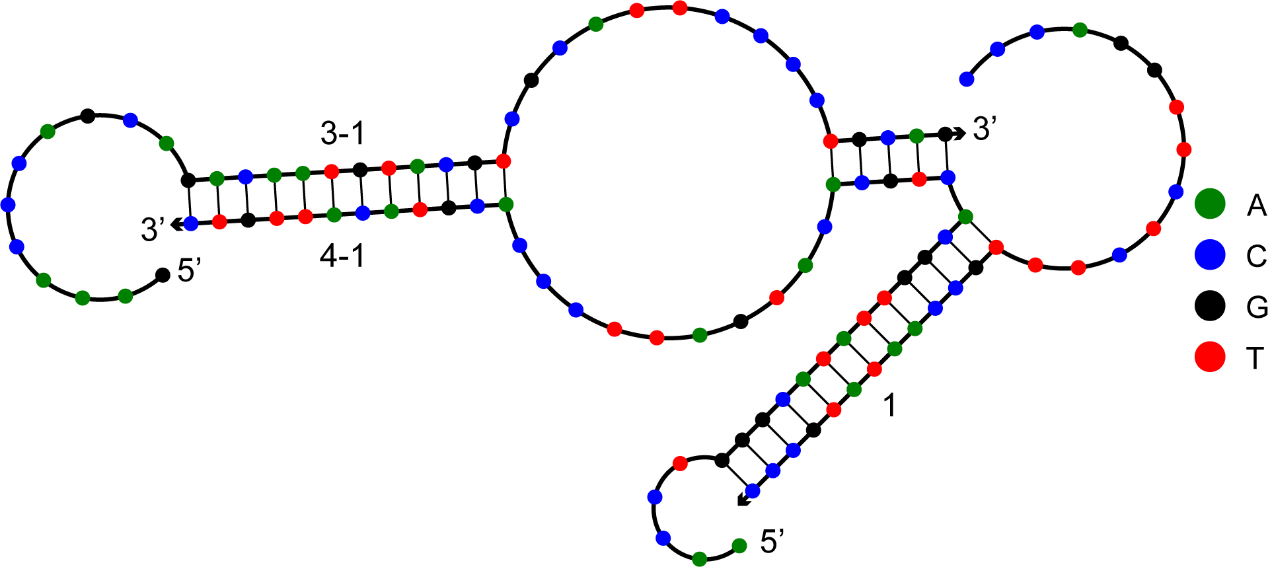


**Figure S1.** NUPACK software was utilized to predict the combination of 1-chain, 3-1-chain, and 4-1-chain at room temperature.


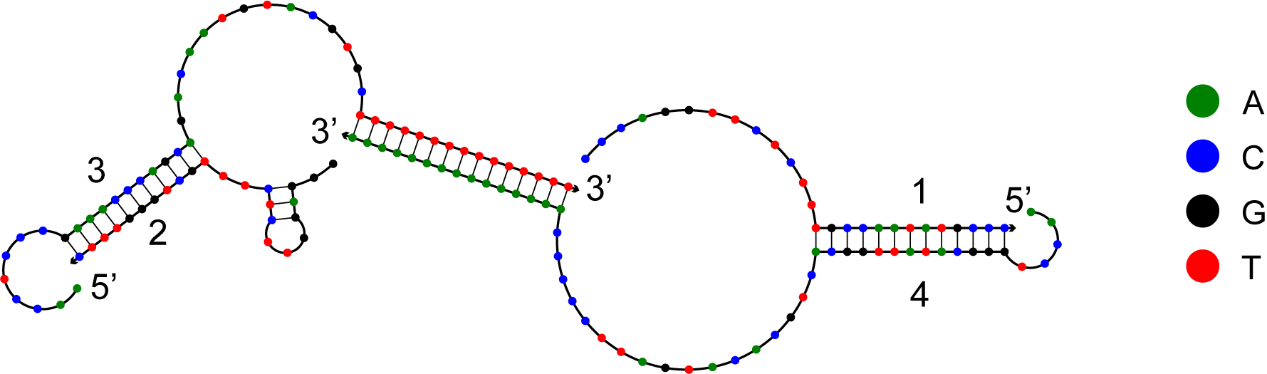


**Figure S2.** Predicted combinations of 1-, 2-, 3- and 4-chain at room temperature using NUPACK software.


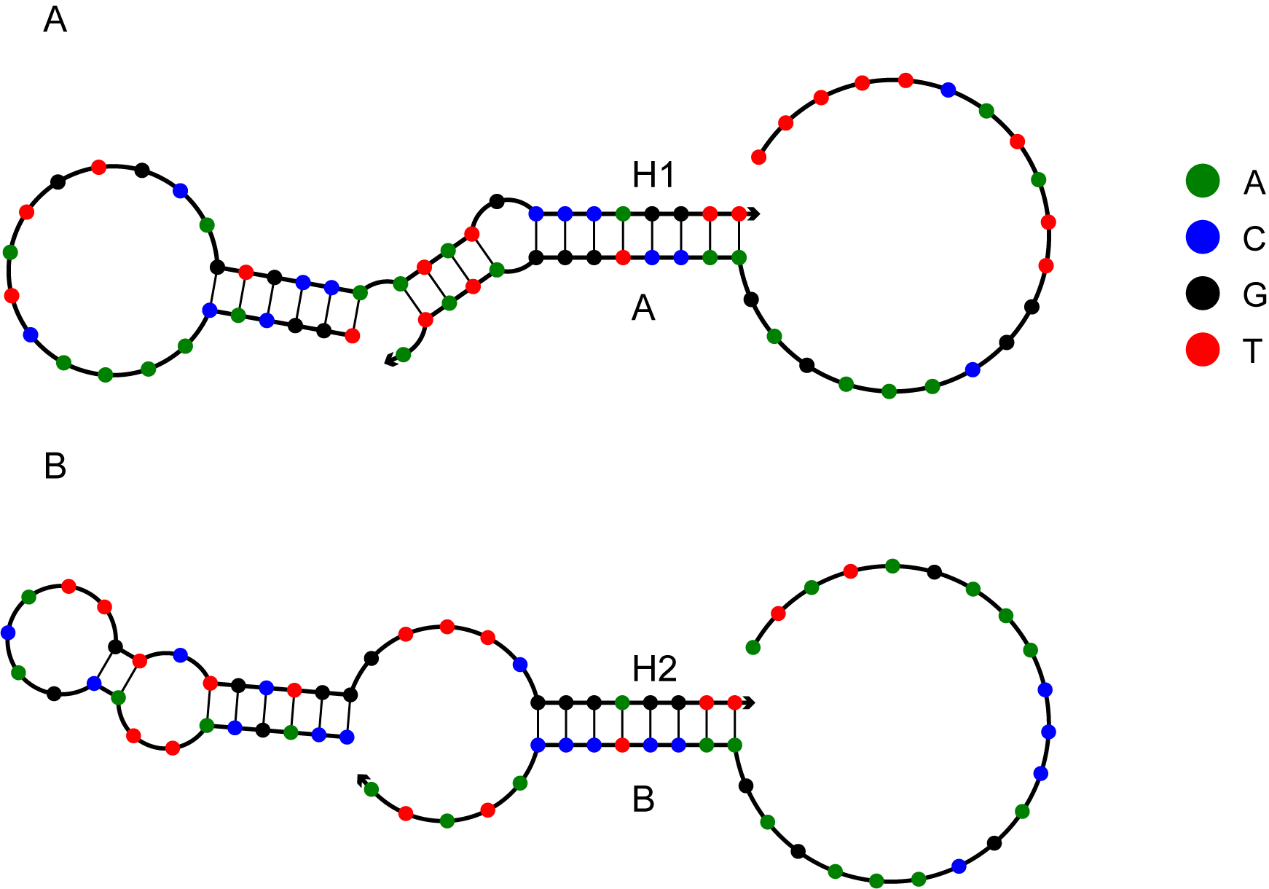
**Figure S3.** Prediction of the combination of AH1 and BH2 chains at room temperature using NUPACK software.


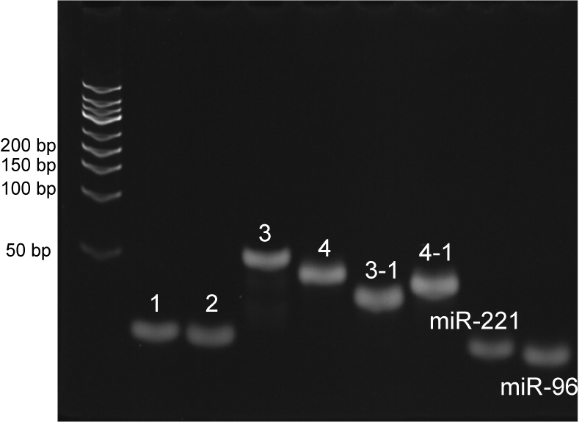


**Figure S4.** Polyacrylamide gel electrophoresis of individual single strands in the DNA logic circuit.


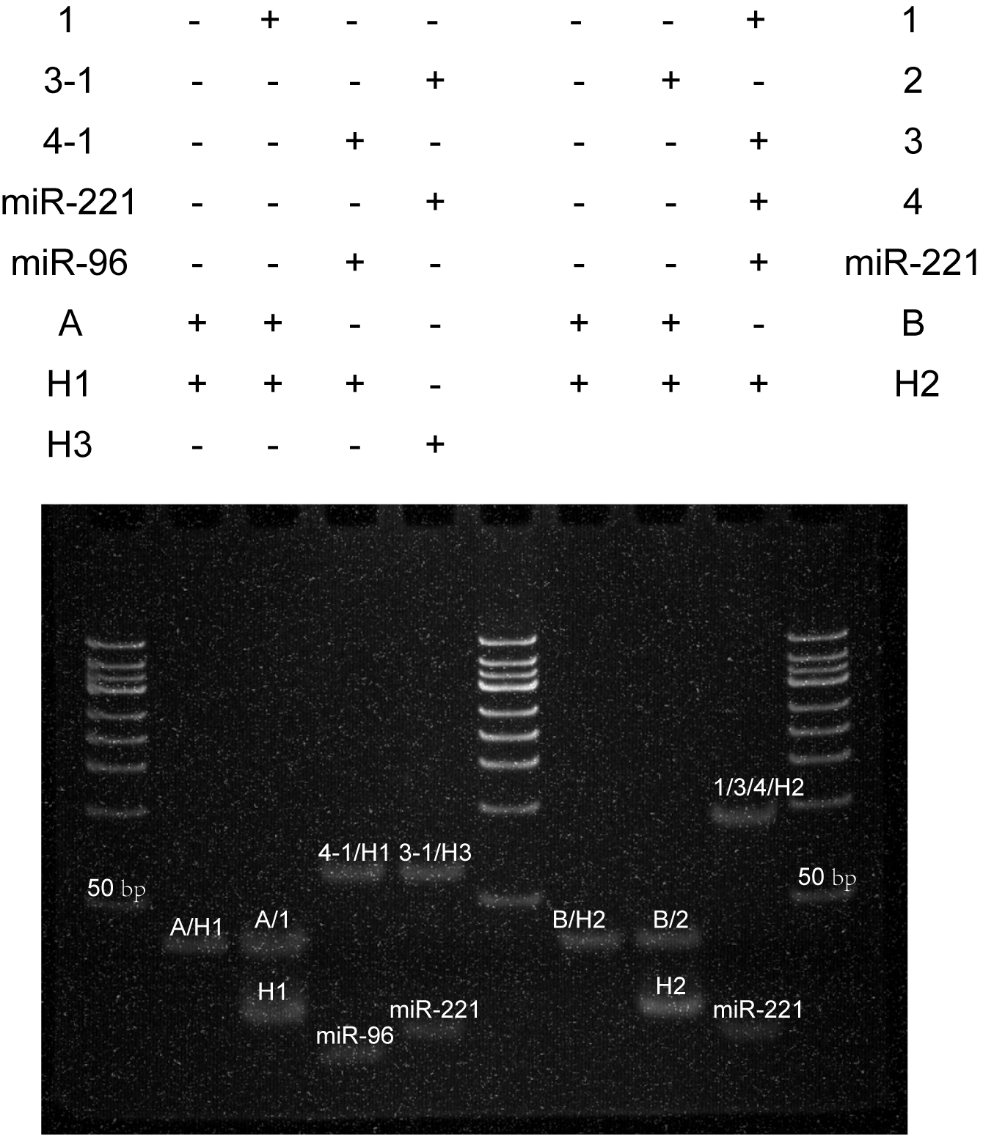


**Figure S5.** Polyacrylamide gel electrophoresis: cyclic chains in logic gates


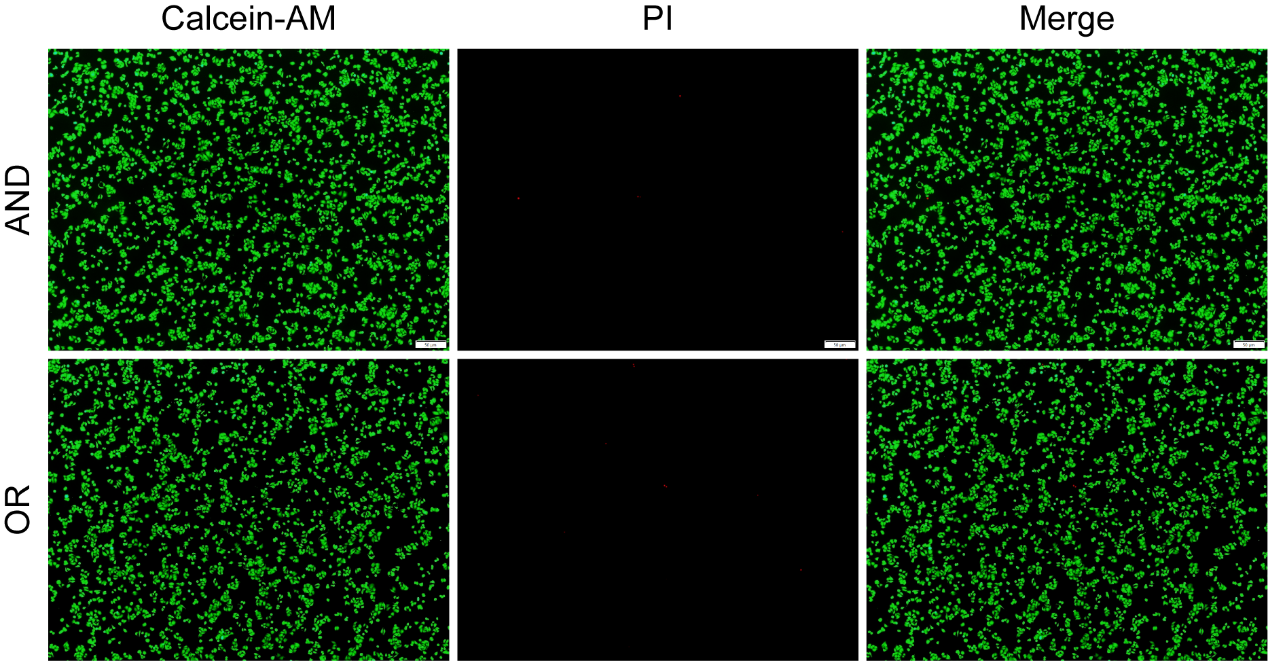


**Figure S6.** Cell live/dead fluorescence imaging of DNA logic circuits after co-incubation with SKOV3 cells. (Scale bar: 50 μm)


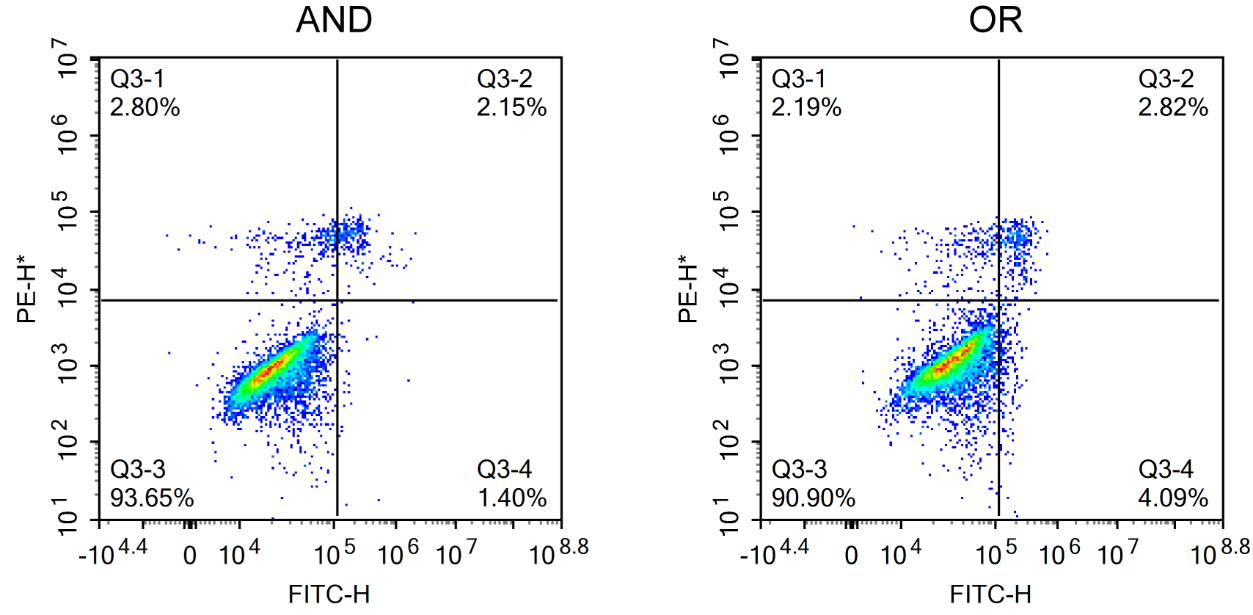


**Figure S7.** Flow cytometry evaluation of DNA logic circuits after co-incubation with SKOV3 cells.


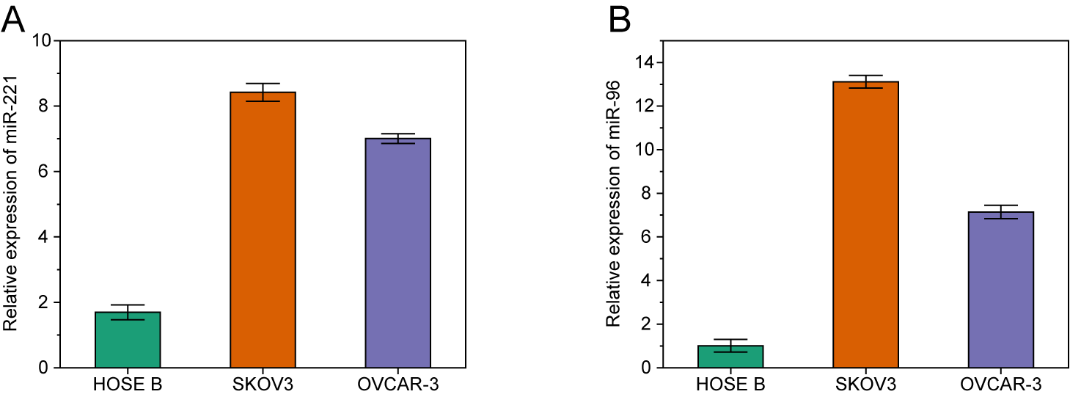


**Figure S8.** qRT-PCR determined the relative levels of miRNAs in different cell species.


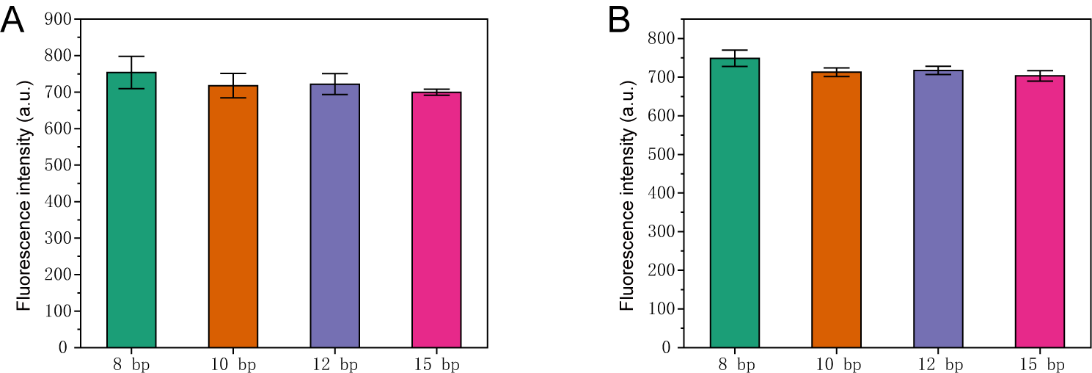


**Figure S9.** In vitro fluorogram. (A) Fluorescence diagram of the final output of “AND” Logic Probe. (B) Fluorescence diagram of the final production of “OR” Logic Probe.


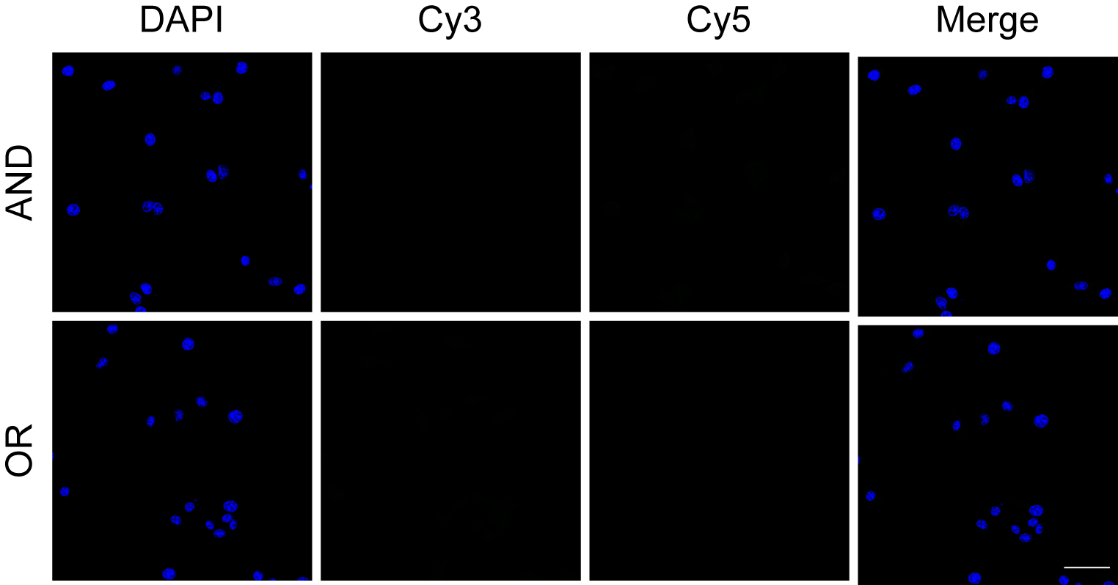


**Figure S10.** Confocal fluorescence imaging of DNA logic circuits after co-incubation with L929 cells. (Scale bar: 60 μm)


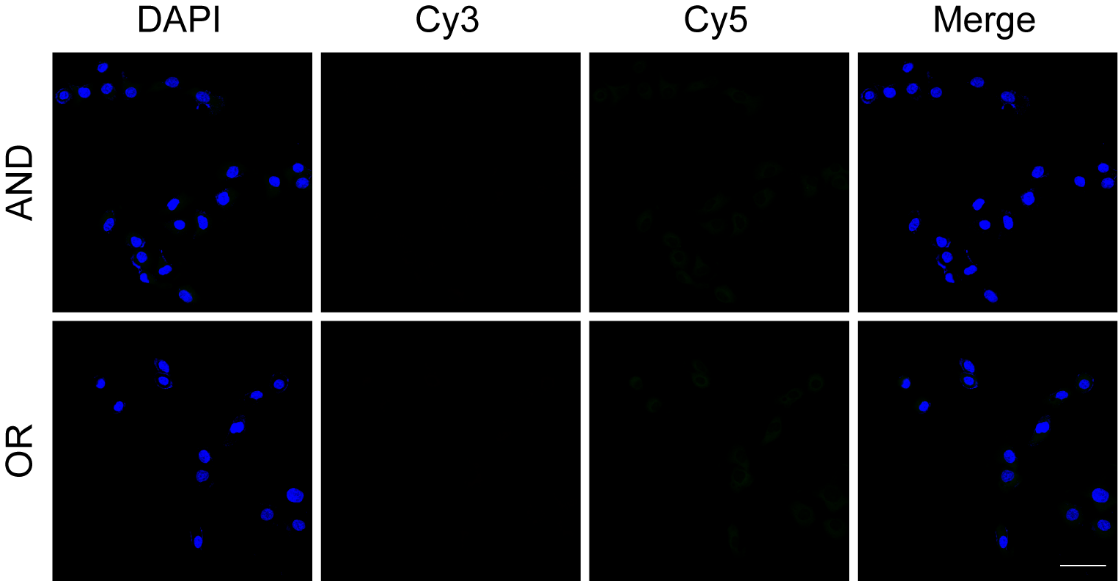


**Figure S11.** Confocal fluorescence imaging of DNA logic circuits after co-incubation with HOSE-B cells. (Scale bar: 60 μm)


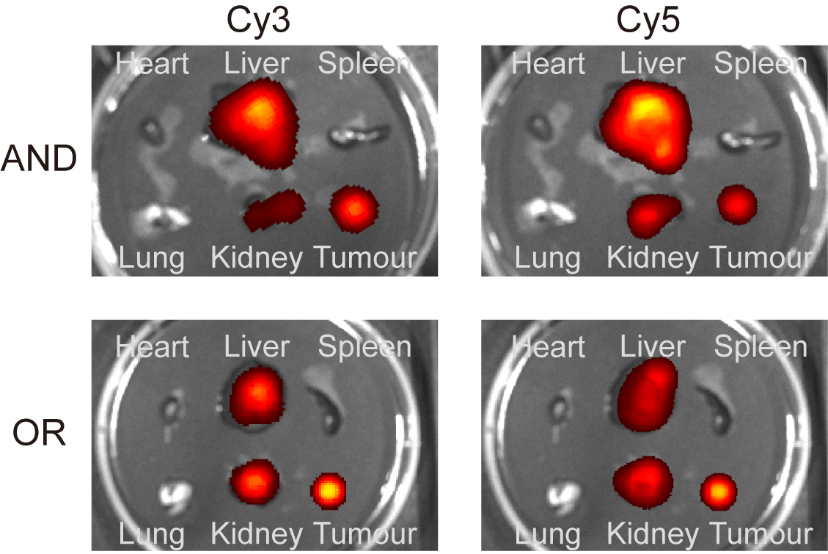


**Figure S12.** SKOV3 tumor-bearing nude mice. Fluorograms of heart, liver, spleen, lung, kidney and tumor after 4 h.


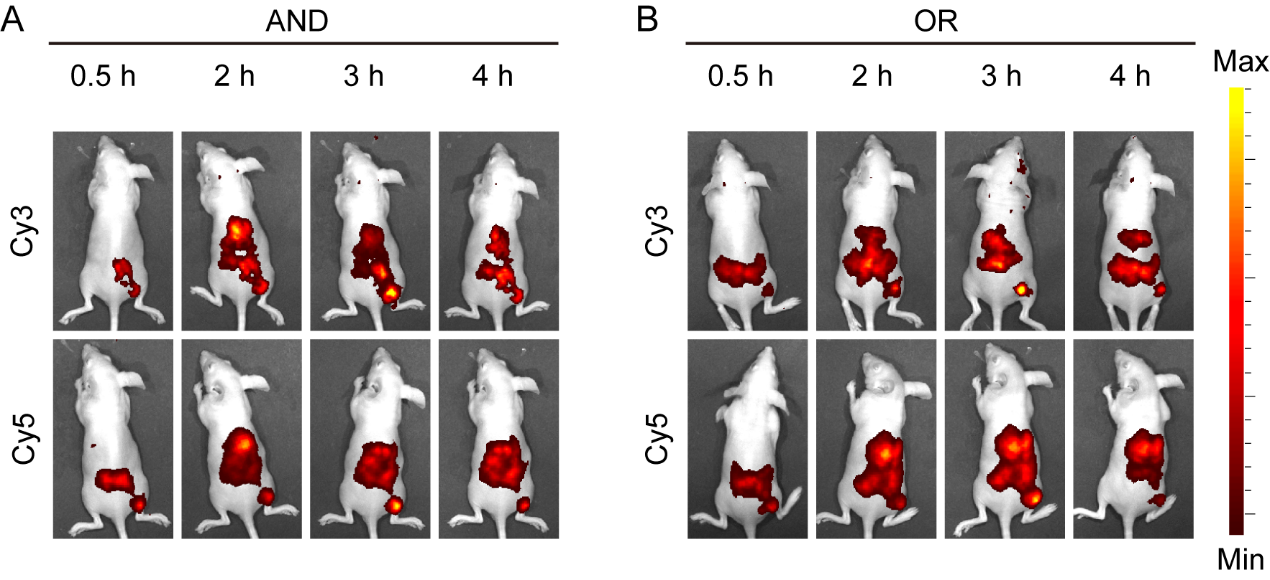


**Figure S13.** OVCAR-3 tumor-bearing nude mice. (A) In vivo fluorescence imaging images at different times after injecting an AND logic circuit into a mouse. (B) In vivo fluorescence imaging maps at different times after injecting OR logic circuits into mice.


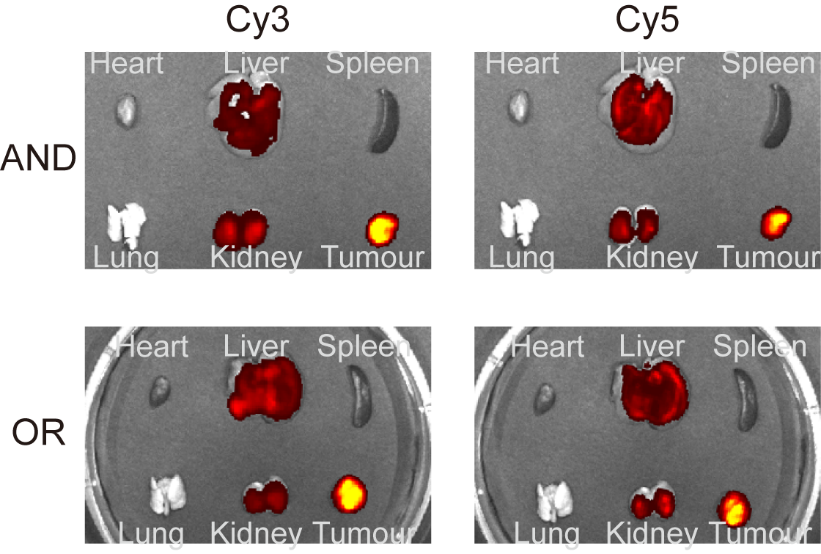


**Figure S14.** OVCAR-3 tumor-bearing nude mice. Fluorograms of heart, liver, spleen, lung, kidney and tumor after 4 h.
